# Automated Facial Recognition for Noonan Syndrome Using Novel Deep Convolutional Neural Network With Additive Angular Margin Loss

**DOI:** 10.3389/fgene.2021.669841

**Published:** 2021-06-07

**Authors:** Hang Yang, Xin-Rong Hu, Ling Sun, Dian Hong, Ying-Yi Zheng, Ying Xin, Hui Liu, Min-Yin Lin, Long Wen, Dong-Po Liang, Shu-Shui Wang

**Affiliations:** ^1^Department of Pediatric Cardiology, Guangdong Provincial People’s Hospital, Guangdong Academy of Medical Sciences, Guangdong Cardiovascular Institute, Guangdong Provincial Key Laboratory of Structural Heart Disease, Guangzhou, China; ^2^Department of Pediatrics, Shantou University Medical College, Shantou, China; ^3^Department of Computer Science and Engineering, University of Notre Dame, South Bend, IN, United States; ^4^Cardiac Center, Guangdong Women and Children Hospital, Guangzhou, China; ^5^Department of Cardiology, Shenzhen Children’s Hospital, Shenzhen, China

**Keywords:** noonan syndrome, facial recognition model, deep learning, Arcface loss function, genetic syndromes

## Abstract

**Background:**

Noonan syndrome (NS), a genetically heterogeneous disorder, presents with hypertelorism, ptosis, dysplastic pulmonary valve stenosis, hypertrophic cardiomyopathy, and small stature. Early detection and assessment of NS are crucial to formulating an individualized treatment protocol. However, the diagnostic rate of pediatricians and pediatric cardiologists is limited. To overcome this challenge, we propose an automated facial recognition model to identify NS using a novel deep convolutional neural network (DCNN) with a loss function called additive angular margin loss (ArcFace).

**Methods:**

The proposed automated facial recognition models were trained on dataset that included 127 NS patients, 163 healthy children, and 130 children with several other dysmorphic syndromes. The photo dataset contained only one frontal face image from each participant. A novel DCNN framework with ArcFace loss function (DCNN-Arcface model) was constructed. Two traditional machine learning models and a DCNN model with cross-entropy loss function (DCNN-CE model) were also constructed. Transfer learning and data augmentation were applied in the training process. The identification performance of facial recognition models was assessed by five-fold cross-validation. Comparison of the DCNN-Arcface model to two traditional machine learning models, the DCNN-CE model, and six physicians were performed.

**Results:**

At distinguishing NS patients from healthy children, the DCNN-Arcface model achieved an accuracy of 0.9201 ± 0.0138 and an area under the receiver operator characteristic curve (AUC) of 0.9797 ± 0.0055. At distinguishing NS patients from children with several other genetic syndromes, it achieved an accuracy of 0.8171 ± 0.0074 and an AUC of 0.9274 ± 0.0062. In both cases, the DCNN-Arcface model outperformed the two traditional machine learning models, the DCNN-CE model, and six physicians.

**Conclusion:**

This study shows that the proposed DCNN-Arcface model is a promising way to screen NS patients and can improve the NS diagnosis rate.

## Introduction

Noonan syndrome (NS) is a genetically heterogeneous disorder with an estimated prevalence of 1 in 1,000–2,500, caused by germline mutations in 11 critical genes of the highly conserved Ras/Mitogen-Activated Protein Kinases (MAPK) pathway (Mendez and Opitz, 1985; Tajan et al., 2018). This multisystem disease is characterized by hypertelorism, ptosis, dysplastic pulmonary valve stenosis, hypertrophic cardiomyopathy, and small stature (Roberts et al., 2013; Li et al., 2019). Early detection and assessment of NS are crucial to formulating an individualized treatment protocol. NS can be diagnosed via clinical features and genetic testing (Van Der Burgt et al., 1994; Roberts et al., 2013). However, because of the complexity and rarity of NS, identifying it remains challenging for pediatric cardiologists and pediatricians. In the wake of this problem, an efficient and convenient auxiliary diagnostic approach is needed for the early diagnosis of NS.

Many genetic syndromes have craniofacial alterations (Hart and Hart, 2009), and facial appearance can be a momentous clue in making an early diagnosis of syndromes (Kuru et al., 2014). The utility of traditional machine learning methods and deep learning methods for diagnosing NS based on pattern recognition of face images has been explored previously by several researchers (Boehringer et al., 2006; Kruszka et al., 2017; Tekendo-Ngongang and Kruszka, 2020; Porras et al., 2021). In 2019, Gurovich et al. (2019) presented a deep DCNN framework, called DeepGestalt, trained on a database of over 17,000 pictures of faces representing more than 200 genetic syndromes. Gurovich et al. (2019) further applied the DeepGestalt model to discriminate five different genotypes of NS and predicted the five desired classes with a top-1 accuracy of 64%. However, to our best knowledge, no studies identified NS patients from healthy children and from children with several other genetic syndromes.

In the present study, therefore, we developed an automated facial recognition model for NS identification based on state-of-the-art tools in the field of facial recognition: a deep convolutional neural network (DCNN) and a novel loss function called Additive Angular Margin Loss (ArcFace; Deng et al., 2019). The main contributions of this study are the following: (1) to our knowledge, this is the first attempt at using a DCNN model with Arcface loss function to generate an automated facial recognition model (DCNN-Arcface model) to identify genetic syndromes; (2) the identification performance of the DCNN-Arcface model outranked two traditional machine learning models; (3) the identification performance of the DCNN-Arcface model was superior to the DCNN framework with cross-entropy loss function (the DCNN-CE model); (4) the identification performance of the DCNN-Arcface model outperformed physicians; and (5) the DCNN-Arcface model can distinguish NS patients from healthy children and from children with several other genetic syndromes.

## Materials and Methods

### Dataset

**FIGURE 1:**
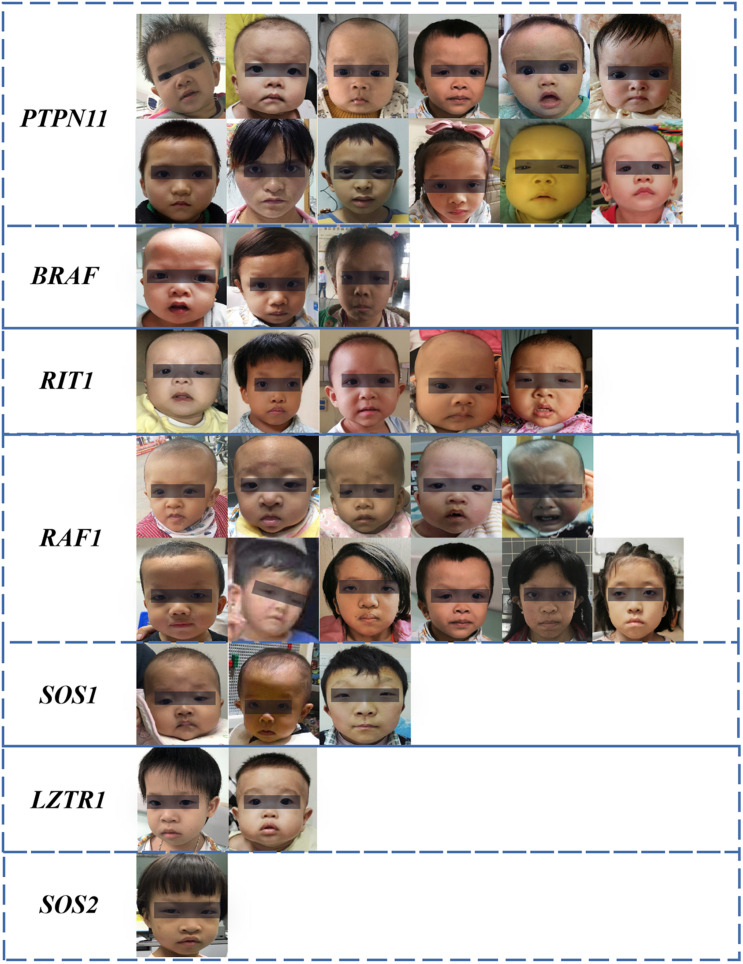
Facial characteristics of NS patients collected from the Guangdong Provincial People’s Hospital, China (*N* = 37). The black bar is used to protect privacy.

The dataset included 127 NS patients (68 males and 59 females), 163 healthy children, and 130 children with several other dysmorphic syndromes (see >Supplementary Table 1). The photo dataset contained only one frontal face image from each participant. Thirty-seven NS patients were recruited from Guangdong Provincial People’s Hospital, between January 2017 and September 2020. Other NS images were obtained from the medical literature (Xu et al., 2017; Leung et al., 2018; Li et al., 2019). The facial characteristics, demographic and genetic characteristics of the NS datasets are summarized in Figure 1 and Tables 1, 2.

**TABLE 1:** Demographic and genetic characteristics of Noonan syndrome patients.

Characteristics	Patients collected from hospital (N = 37)	Patients collected from medical literature (N = 90)
Female sex, *n* (%)	23 (62.1)	36 (40.0)
Age period when face images were taken, *n* (%)		
Infancy (1–12 months)	12 (32.4)	44 (48.9)
Childhood (1–12 years)	21 (56.8)	43 (47.8)
Adolescence (12–18 years)	4 (10.8)	3 (3.3)
Type of gene mutations, *n* (%)		
*PTPN11*	12 (32.4)	47 (52.2)
*BRAF*	3 (8.1)	2 (2.2)
*KRAS*	0 (0.0)	13 (14.4)
*LZTR1*	2 (5.4)	1 (1.1)
*RAF1*	11 (29.7)	10 (11.1)
*RTI1*	5 (13.5)	9 (10.0)
*NRAS*	0 (0.0)	1 (1.1)
*SOS1*	3 (8.1)	7 (7.8)
*SOS2*	1 (2.6)	0 (0.0)

**TABLE 2:** Pathogenic variants detected in Noonan syndrome patients collected from the Guangdong Provincial People’s Hospital.

Gene	DNA change	Protein change	Number of times observed	Origin of Mutation
*PTPN11*	c.922A > G	p.N308D	3	*de novo*
	c.188A > G	p.Y63C	1	de novo
	c.124A > G	p.T42A	1	de novo
	c.1492C > T	p.R498W	2	de novo
	c.1528C > G	p.Q510E	3	de novo
	c.1517A > C	p.Q506P	1	de novo
	c.174C > G	p.N58K	1	de novo
BRAF	c.1502A > G	p.E501G	1	de novo
	c.1796C>G	p.T599R	1	de novo
	c.736G > C	p.A246P	1	de novo
LZTR1	c.2098A>G	p.M700V	1	de novo
	c.1291G > A	p.E431K	1	de novo
RAF1	c.770C > T	p.S257L	6	de novo
	c.775T > A	p.S259T	3	de novo
	c.1082G > C	p.G361A	1	de novo
	c.781C > A	p.P261T	1	de novo
RIT1	c.170C > G	p.A57G	1	de novo
	c.229G > A	p.A77T	1	de novo
	c.284G > C	p.G95A	1	de novo
	c.246T > A	p.F82L	1	de novo
	c.270G > C	p.M90L	1	de novo
SOS1	c.508A>G	p.K170E	1	de novo
	c.1654A > G	p.R552G	1	de novo
	c.2536G > A	p.E846K	1	de novo
SOS2	c.1502A > G	p.E501G	1	de novo

All face images in this study were required to fulfill two eligibility criteria. First, the diagnosis of NS and other genetic syndromes was confirmed by fluorescence in situ hybridization, karyotype analysis or next-generation sequencing. Second, the faces should be sufficiently legible and oriented.

### The DCNN-Arcface Model

**FIGURE 2:**
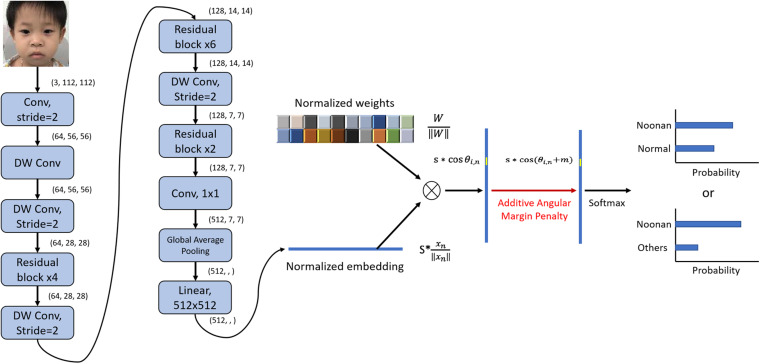
Architecture of DCNN-Arcface model for Noonan syndrome identification. We used convolutional layers with stride = 2 instead of max-pooling to half the feature map size and double channels number. After extracting the embeddings with multiple convolutional layers, we normalized the weights of the last fully connected layer ∥*W*_*i*_∥ = 1 with L_2_ normalization and rescaled the norm of embedding vector to s, ∥χ_*n*_∥ = *s*. Then, an angular margin penalty m was added to the target angle θ_l,m_. After that, *cos*⁡(θ_*l*,*m*_ + m) was calculated, and all logits were multiplied by the feature scale s. The logits then went through the SoftMax function to derive the probability for each class. “DW conv” represents depth-wise convolution.

The architecture of the DCNN-Arcface model is illustrated in Figure 2.

#### Image Preprocessing

**FIGURE 3:**
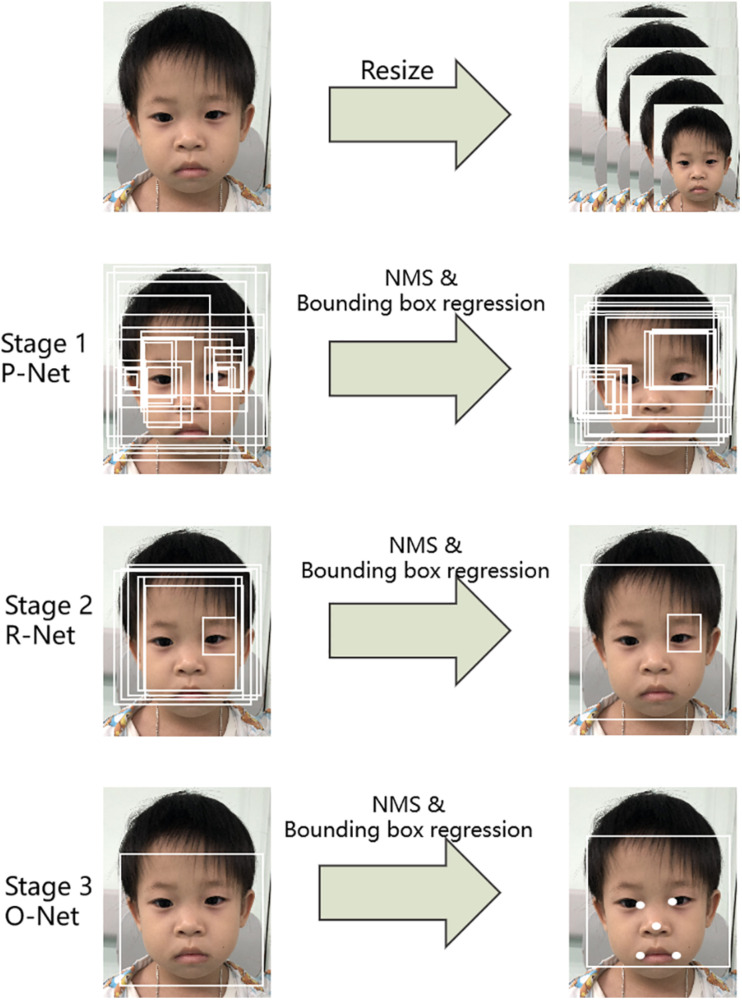
Pipeline of the multi-task convolutional neural network (MTCNN).

The first step was to detect the patient’s face in an input image. Here, a multi-task convolutional neural network (MTCNN; Zhang et al., 2016) was applied to detect face image areas. It ultimately utilized five facial landmarks (Figure 3). The MTCNN contained an image pyramid and a three-stage cascaded framework. When a raw face image was given, different scale ratios were used to resize the face image to build an image pyramid. It was then transmitted to the three-stage cascaded framework as an input. After training on three convolutional networks [proposal network (P-Net), refine network (R-Net), and output network (O-Net)], the image pyramid was finally converted into five facial landmark positions. We subsequently cropped the face image containing the five facial landmarks into (3, 112, 112).

#### Feature Extraction and Image Embedding Using a Novel Deep Convolutional Neural Network

**FIGURE 4:**
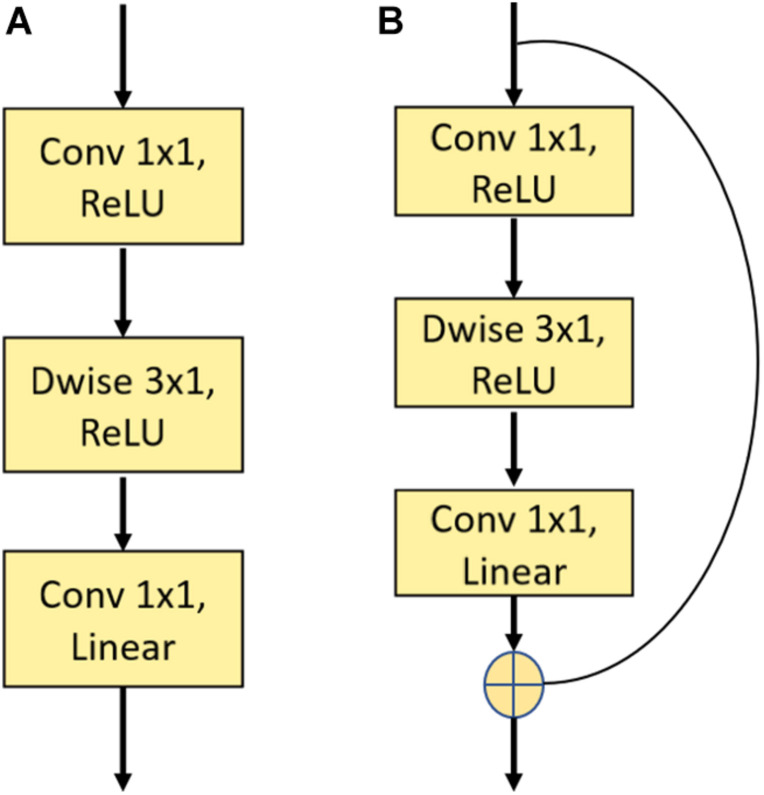
Illustration of a single depth-wise convolution block and residual block. **(A)** The construction of the Depth-wise (Dwise) convolution block denoting a convolutional layer with a convolution group number set as input channels. **(B)** The construction of the residual block. “Linear” means that there is no use of an activation function.

In this work, we used a novel two-dimension DCNN to extract face features and then embed the face images in N-dimensional vector space *x*_*n*_ ∈ ℜ^*N*^. The first layer was a traditional convolution block with a kernel size of 7 × 7, which was followed by multiple residual blocks (He et al., 2016) and depth-wise convolution blocks (Bai et al., 2018). Figure 4 depicts the construction of a single residual block and depth-wise (Dwise) convolution block. The Dwise convolution block consisted of three convolutional operations followed by a nonlinear unit, the Rectified Linear Unit (ReLU). The construction of the residual block was similar to the Dwise block, except for the addition of “shortcut connections” to feedforward neural networks. After multiple layers of feature extraction, we used a global average pooling layer to flatten the feature maps followed by a fully connected layer. The input face images were finally embedded in N-dimension vector space *x*_*n*_∈ℜ^*N*^.

#### Arcface Loss Function

In this work, we used a loss function representing state-of-the-art in the field of facial recognition, called ArcFace loss function (Deng et al., 2019). The main idea is based on the observation that the weight matrix from the last fully connected layer can be viewed as a combination of vectors that represent the conceptual centers corresponding to different face classes. In detail, after extracting the embeddings with multiple convolutional layers, we rescaled the norm of the embedding vector to *s*, ∥χ_*n*_∥ = *s*, where x_n_represents the embedding vector of the *n*th sample. Then, the individual wights of the last fully connected layer were normalized with L_2_ normalization, ∥*W*_*i*_∥ = 1, in which W_i_ is the weight of the fully connected layer of class *i*. Thus, the product $W_{i}^{\text{T}}*\chi_{n}$ reflected the angle between the embedding vector and the weight of class *i* in the fully connected layer.

The loss function was then defined as,

(1) $$L_{A}=-\sum_{n}\omega_{i}\,\log\frac{e^{∥W_{i}^{T}∥\,∥x_{n}∥\,\cos\theta_{i,n}}}{\sum_{j}e^{∥W_{J}^{\text{T}}∥\,∥x_{n}∥\,\cos\theta_{j,n}}}$$

where ω_*i*_is the weight of class *i* to deal with biased dataset. In our case, since our dataset has balanced of patient for each class, we set all ω_*i*_ as 1.

By minimizing the loss function, embedding vectors for images within the same class were forced to gather around the same weight vector in the vector space, which enhanced intra-class compactness. Moreover, an additive angle margin *m* was added to improve the robustness and discrepancy for different classes, according to Deng et al. (2019).

(2) $$L_{A}=-\sum_{n}\omega_{i}\,\log\frac{e^{s*\cos\left(\theta_{i,n}+m\right)}}{e^{s*\cos\left(\theta_{i,n}+m\right)}+\sum_{j\neq i}e^{s*\cos\theta_{j,n}}}$$

Mathematically, the positive additive angle decreases the value of cos (θ_i,  n_ + *m*). If we try to maintain the value of the overall loss function, we can either reduce θ_i,  n_ or increase θ_j,  n_. That is, we can bring the embedding vector closer to the weight vector of the same class or further away from other classes.

#### Setting

We first pre-trained our network on a public human face dataset, CASIA (Wen et al., 2016), and then retrained the whole network on the NS identification task. In the training process, we applied rotation and horizontal flipping to augment the dataset. Each face image was rotated at angles θ = [90°, 180°] and horizontally flipped. Thus, three face images were generated for each original face image. We used Adam as our optimizer, and the learning rate was set to 1e^–4^. Five-fold cross-validation was performed to evaluate the performance of DCNN-Arcface model, DCNN-CE model, and two traditional machine learning models. The proportion of training set, validation set, and test set was 3:1:1. For the DCCN-Arcface model, in the testing phase, we calculated the embeddings for all faces in the training set and derived the average embedding for each class as a reference vector. We used the angle between the inference image’s embedding vector and the reference vector for prediction instead of the weights on the last fully connected layer (Deng et al., 2019).

### Comparative Experiments

In this study, we implemented a DCNN-CE model and two traditional machine learning models. Comparisons of the proposed DCNN-Arcface model with these three models were performed.

#### Comparison With Traditional Machine Learning Models

**FIGURE 5:**
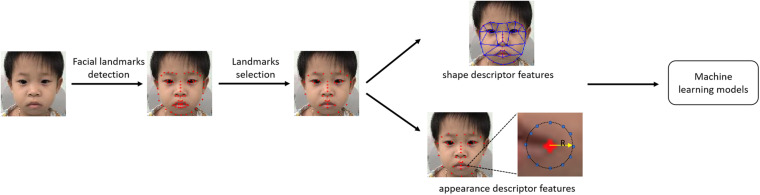
Illustration of two traditional machine learning models.

Before the prevalence of deep learning methods, various supervised machine learning models were used for NS identification (Boehringer et al., 2006; Kruszka et al., 2017). Therefore, we applied two supervised traditional machine learning methods to construct two additional facial recognition models for NS identification: a support vector machine (SVM) (SVM-Linear model) and logistic regression (LR) (LR model). These methods were unable to take image data as input directly. Therefore, the first step was to extract features from patients’ faces. We used a landmark detector from the “dlib” Python library to locate 68 informative points from each patient’s face, including the outline of the eyebrows, eyes, nose, mouth, and jaw. The landmark detector was an implementation of Kazemi and Sullivan (2014), in which an ensemble of regression trees is trained with manually labeled data to estimate the coordinates of facial landmarks. Then, two types of features—shape descriptor features and appearance descriptor features—were measured based on those landmarks. We calculated the distances between every two points as the shape information, although this was more complicated for the appearance descriptor feature. For each point, we derived the local binary pattern (LBP) with neighboring sample points set at 12 and the radius set at 4. Thus, we could compute six statistics for the LBP histogram: the mean, variance, skewness, kurtosis, energy, and entropy. We found that some landmarks were within a small area, such that features extracted from those points may provide redundant information for identification. Therefore, we selected 38 landmarks for feature measurement. The total number of features comprised 703 shape descriptor features and 266 appearance descriptor features. The concatenated feature vectors were then sent to the SVM with a linear kernel and to LR for NS identification. Feature selection was based on each feature’s weight in the classification model, and we treated smaller weights as being less significant to the identification task. We tried different numbers of features from 10 to 969. We found that the area under the ROC curve started converging with 400 for identifying NS patients from healthy children, and 300 features for identifying NS patients from children with other genetic syndromes. The detailed architecture is shown in Figure 5.

#### Comparison With the DCNN-CE Model

Cross-entropy (Rubinstein, 1999) loss function is the most widely used loss function for classification problems. It can provide a precise mathematical framework with high speed (Boer et al., 2005). Hence, we constructed another DCNN-based face recognition model of NS. The same DCNN framework were used, but Arcface loss function was substituted by the cross-entropy loss function.

### Comparison With Physicians

In this study, we performed two experiments to determine the identification performance by six physicians with different areas of expertise (two pediatricians, two pediatric cardiologists, and two clinical geneticists). To distinguish NS patients from healthy children, the physicians were presented with a dataset consisting of all NS patients and healthy children in random order. They classified these face images as either NS patients or healthy children. Likewise, after rearranging all photographs of the NS patients and those of children with several other genetic syndromes, the physicians classified these face images as NS patients or children with several other genetic syndromes. Each face image was shown for 10 s without exhibiting any clinical data. The experiments were repeated three times.

### Evaluation Metric

The metrics we used to evaluate the identification performance in all comparative experiments were the total identification accuracy measure, sensitivity measure, and specificity measure. Moreover, the area under the receiver operating characteristic curve (AUC) and the area under the precision–recall curve (AP scores) were also determined. These measures were calculated as follows:

$$T\,o\,t\,a\,l\,A\,c\,c\,u\,r\,a\,c\,y=\frac{T\,P+T\,N}{A\,m\,o\,u\,n\,t\,o\,f\,\text{all}\,\text{images}}$$

$$S\,e\,n\,s\,i\,t\,i\,v\,i\,t\,y=\frac{T\,P}{T\,P+F\,N}$$

$$S\,p\,e\,c\,i\,f\,i\,c\,i\,t\,y=\frac{T\,N}{T\,N+F\,P}$$

where TP, TN, FP, and FN denote true positives, true negatives, false positives, and false negatives, respectively.

All measurements are reported as the mean ± standard deviation. These measurements indicated the ability of all methods to correctly distinguish NS patients from healthy children and children with several other genetic syndromes.

### Statistical Analysis

McNemar’s test was used to determine the disagreement for binary outputs between DCNN-Arcface model and DCNN-CE model, DCNN-Arcface model and traditional machine learning models (Dietterich, 1998). Z-tests were constructed to compare the AUC and AP scores of DCNN-Arcface model, DCNN-CE model, and two machine learning models (Zhang et al., 2002). *p*-values < 0.05 were considered statistically significant.

## Results

### Accuracy, Specificity, Sensitivity, AUC, and AP Score of Different Models

Tables 3, 4 list the accuracy, specificity, sensitivity, AUC, and AP score of the proposed DCNN-Arcface model, DCNN-CE model, SVM-linear model, and LR model.

**TABLE 3:** The accuracy, specificity, sensitivity, AUC, and AP score of different models at distinguishing Noonan syndrome from healthy children.

models	Accuracy (mean ± SD)	Specificity (mean ± SD)	Sensitivity (mean ± SD)	AUC (mean ± SD)	AP score (mean ± SD)
DCNN-Arcface	**0.9201 ± 0.0138**	**0.9774 ± 0.0120**	**0.8381 ± 0.0208**	**0.9797 ± 0.0055**	**0.9801 ± 0.0145**
DCNN-CE	0.8521 ± 0.0207	0.8744 ± 0.0362	0.8201 ± 0.0072	0.9357 ± 0.0085	0.9267 ± 0.0170
SVM-linear	0.8259 ± 0.0210	0.8343 ± 0.0245	0.8138 ± 0.0170	0.9031 ± 0.0064	0.9020 ± 0.0086
LR	0.7877 ± 0.0109	0.8363 ± 0.0160	0.7184 ± 0.0050	0.8669 ± 0.0035	0.8636 ± 0.0021

*Values in bold indicate the optimal performance. SD, standard deviation.*

**TABLE 4:** The accuracy, specificity, sensitivity, AUC, and AP score of different models at distinguishing Noonan syndrome from patients with several other genetic syndromes.

models	Accuracy (mean ± SD)	Specificity (mean ± SD)	Sensitivity (mean ± SD)	AUC (mean ± SD)	AP score (mean ± SD)
DCNN-Arcface	**0.8171 ± 0.0074**	**0.9477 ± 0.0116**	**0.7794 ± 0.0252**	**0.9274 ± 0.0062**	**0.9356 ± 0.0067**
DCNN-CE	0.7848 ± 0.0205	0.7907 ± 0.0155	0.6960 ± 0.0207	0.8594 ± 0.0106	0.8739 ± 0.0108
SVM-linear	0.7048 ± 0.0190	0.6982 ± 0.019	0.7112 ± 0.0049	0.7627 ± 0.0161	0.7499 ± 0.0257
LR	0.7210 ± 0.0111	0.7273 ± 0.0407	0.7150 ± 0.0294	0.7694 ± 0.0102	0.7467 ± 0.0025

*Values in bold indicate the optimal performance. SD, standard deviation.*

### Comparison With Traditional Machine Learning Models

**FIGURE 6:**
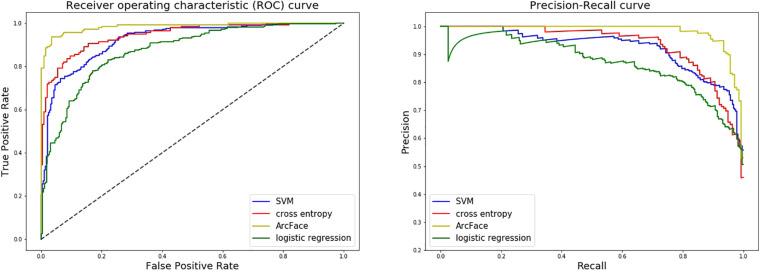
ROC curves and P–R curves of four different models when distinguishing children with Noonan syndrome from healthy children. The DCNN-Arcface model is consistently better than the other three.

**FIGURE 7:**
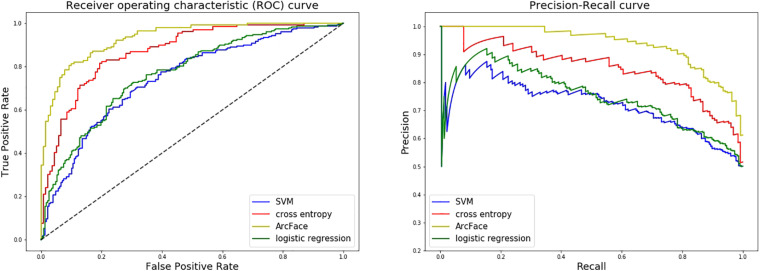
ROC curves and P–R curves of four different models when distinguishing children with Noonan syndrome from those with several other genetic syndromes. The DCNN-Arcface model is consistently better than the other three.

At distinguishing NS patients from healthy children, the DCNN-Arcface model achieved the best identification performance compared with the SVM-Linear model (*p* = 0.0002, McNemar’s test) and with the LR model (*p* = 0.0001, McNemar’s test). At distinguishing NS patients from children with several other genetic syndromes, the performance of the DCNN-Arcface model was also superior to the SVM-Linear model (*p* = 0.0001, McNemar’s test) and the LR model (*p* = 0.0000, McNemar’s test). The ROC curves and precision–recall (P–R) curves of different models with different tasks are shown in Figures 6, 7. The highest AUC and AP scores were achieved by the DCNN-Arcface model on both tasks (*p* = 0.0000, z-test). The ROC curves and P–R curves shown in Figures 6, 7 also indicate that the DCNN-Arcface model can significantly improve the identification performance of an NS facial recognition model.

### Comparion With the DCNN-CE Model

At distinguishing NS patients from healthy children, the performance of the DCNN-Arcface model outranked the DCNN-CE model (*p* = 0.0016, McNemar’s test). At distinguishing NS patients from children with several other genetic syndromes, better performance was also obtained from the DCNN-Arcface model (*p* = 0.0045, McNemar’s test). Significantly statistical difference was achieved when compare the AUC and AP score obtained from the DCNN-Arcface model with that of the DCNN-CE model (*p* = 0.0000, z-test). The ROC curves and P–R curves shown in Figures 6, 7 also elucidated the improved identification performance of NS facial recognition by applying the DCNN-Arcface model.

### Comparison With Physicians

The identification performance of different physicians for different tasks is presented in Tables 5, 6. All six physicians completed the identification tasks, with an average accuracy of 0.7595 ± 0.0618 for identifying NS patients from healthy children, and 0.5826 ± 0.0303 for identifying NS patients from children with several other genetic syndromes. When classifying the physicians depend on their expertise, the clinical geneticists exhibited the best identification performance among the physicians, with the average accuracy of 0.7685 ± 0.1223 for identifying NS patients from healthy children, and 0.6105 ± 0.0106 for identifying NS patients from children with several other genetic syndromes. However, the DCNN-Arcface model outperformed all six physicians according to all metrics on both tasks, including accuracy, specificity, and sensitivity (Tables 3, 4).

**TABLE 5:** Identification performance of different physicians at distinguishing Noonan syndrome patients from healthy children.

Levels	Accuracy (mean ± SD)	Specificity (mean ± SD)	Sensitivity (mean ± SD)
Pediatrician (*N* = 2)	0.7515 ± 0.0389	0.7880 ± 0.0212	0.7045 ± 0.1167
Pediatric cardiologists (*N* = 2)	0.7585 ± 0.0488	**0.8555 ± 0.1690**	0.6335 ± 0.3288
Clinical geneticists (*N* = 2)	**0.7685 ± 0.1223**	0.7685 ± 0.1973	**0.7750 ± 0.0170**

*Values in bold indicate the optimal performance. SD, standard deviation.*

**TABLE 6:** Identification performance of different physicians at distinguishing Noonan syndrome patients from children with several other genetic syndromes.

Levels	Accuracy (mean ± SD)	Specificity (mean ± SD)	Sensitivity (mean ± SD)
Pediatrician (*N* = 2)	0.5640 ± 0.0382	0.4535 ± 0.0431	**0.6770 ± 0.0339**
Pediatric cardiologists (*N* = 2)	0.5735 ± 0.0247	**0.7640 ± 0.2687**	0.3855 ± 0.2340
Clinical geneticists (*N* = 2)	**0.6105 ± 0.0106**	0.7460 ± 0.1739	0.4725 ± 0.2001

*Values in bold indicate the optimal performance. SD, standard deviation.*

## Discussion

Distinctive facial appearance provides significant information for predicting a specific genetic syndrome (Roosenboom et al., 2016). NS’s facial gestalt usually includes a high forehead, low posterior hairline, hypertelorism, highly arched palate, downslanting palpebral fissures, epicantal folds, ptosis, high wide peaks of the vermilion, deeply grooved philtrum, and low-set and posteriorly rotated ears (Tartaglia et al., 2011; Li et al., 2019). The diagnosis of NS could start with astute clinicians recognizing the specific facial dysmorphism. As they age, however, the faces of children with NS can become more atypical, as the face lengthens and becomes more triangular in shape (Allanson, 2016). Some genotypes of NS present with atypical facial characteristics (Jenkins et al., 2020). In addition, the facial appearance of NS is similar to that of other RASopathies, such as Cardio-faciocutaneous and Costello syndromes (Allanson, 2016). In this context, discriminating this particular syndrome based on facial appearance is challenging, and can lead to misdiagnose and misclassification.

In 2003, Loos et al. (2003) first used Gabor wavelet transformation, a machine learning method, to classify five syndromes with an accuracy of 76%. Machine learning is a subfield of Artificial Intelligence that allows computers to learn from data to make predictions for a given task without being explicitly programmed (Nguyen et al., 2019). Since then, other traditional machine learning methods have been reported for NS facial recognition. In 2006, Boehringer et al. (2006) first used principal component analysis to reduce covariates from face images of NS patients, and then applied linear discriminant analysis, SVM and *k-*th nearest neighbors to discriminate NS from other genetic syndromes with a maximum accuracy of 79.4%. In 2017, Kruszka et al. (2017) applied the independent component analysis to locate facial landmarks, and subsequently used a linear support vector machine as a classifier to classify NS patients and healthy children. They achieved sensitivites and specificities for Caucasian, African, Asian, and Latin American children of 0.95 and 0.93, 0.94 and 0.91, 0.95 and 0.90, and 0.96 and 0.98, respectively (Kruszka et al., 2017). Recently, Porras et al. (2021) applied the method presented by Kruszka et al. (2017) to discriminate patients with NS from those with Williams-Beuren syndromes, and obtained an accuracy of 85.68%. In the above methods, after extracting features with traditional machine learning methods, feature selection is usually performed to accelerate the relevancy and prevent over-fitting. However, both of these steps are complicated and time-consuming (Wang et al., 2018). With this limitation, the computer vision community has shifted toward DCNNs for medical image classification tasks (Zhang et al., 2019).

A DCNN is a feed-forward artificial neural network that consists of multiple convolutional layers followed by a nonlinear unit (Ongsulee, 2017). It can automatically learn representation from labeled data without requiring human expertise for feature extraction (Wang et al., 2018). Complicated layers are often constructed to achieve more satisfactory accuracy in facial recognition tasks (He et al., 2016). However, accuracy may be saturated and it can degrade rapidly as the network becomes deeper. In 2016, He et al. (2016) introduced a deep residual learning framework to solve this degradation problem by adding a residual block. The residual block mainly involved a “shortcut connection” that denotes the outputs of the identity mapping added to the outputs of the stacked layers. With this residual block, complicated neural networks are more easily trained (He et al., 2016). Nevertheless, the deep residual learning framework is still computationally expensiveness with respect to its size and content. A depth-wise convolution block can lower this computational complexity. Depth-wise convolution splits the standard convolution into two separate layers for filtering and combining. Through this optimization, the convolutional operation becomes more efficient in terms of the number of parameters and the computational cost (Bai et al., 2018). In the present study, we combined the residual block and the depth-wise block to develop a novel DCNN framework. The joint combination of these two blocks enabled us to build a light architecture without sacrificing accuracy.

The loss function guides the DCNN to extract features from input images by backpropagating gradients to the weights in the network. The definition of a loss function can impact the discriminative ability of a model. Cross-entropy is the most widely used loss function for classification tasks. However, cross-entropy fails to teach the neural network the similarity among samples belonging to the same class (Deng et al., 2019). The ArcFace is a novel loss function developed by Deng et al. (2019). By adding an additive angular margin penalty between deep features and the ground truth weight, Arcface loss function can simultaneously enhance the intra-class compactness compared to cross-entropy (Deng et al., 2019). Chinapas et al. (2019) adapted Arcface loss function to train personal verification system and achieved a maximum accuracy of 0.996. However, to the best of our knowledge, this loss function has not been used for genetic syndrome facial recognition tasks. NS has 12 different genotypes, and some of the genotypes present with an atypical facial appearance (Allanson et al., 2010). Due to this intrinsic intra-class variation, learning the discriminative features of NS is challenging. Arcface loss function addresses this problem with its distinctive ability for compact intra-class variation. Hence, it is more suitable for NS identification.

In the present study, we implemented the DCNN framework and Arcface loss function to construct an automated facial recognition model for NS identification. The DCNN-Arcface model achieved an accuracy of 0.9201 ± 0.0138 and an AUC of 0.9797 ± 0.0055 when distinguishing NS patients from healthy children, and an accuracy of 0.8171 ± 0.0074 and an AUC of 0.9274 ± 0.0062 when distinguishing NS patients from children with several other genetic syndromes. It outperformed all six physicians in terms of accuracy, sensitivity, and specificity. NS usually presents with considerable heterogeneity in clinical manifestations (Roberts et al., 2013), and it is a rare syndrome. As such, prompt diagnosis of NS in routine clinical practice is still a cumbersome problem for physicians. Although previous literature has shown that the DCNN-based facial recognition models can assist in diagnosing genetic syndromes (Gurovich et al., 2019; Qin et al., 2020), only a few studies have used DCNNs to identify NS. In 2020, Tekendo-Ngongang and Kruszka (2020) applied DeepGestalt, a DCNN-based architecture, to develop a NS facial recognition model. Their model discriminated NS patients from matched healthy individuals with an AUC of 0.979. However, the DeepGestalt model used cross-entropy as a loss function. By using a novel DCNN framework and Arcface, our DCNN-Arcface model can efficiently discriminate NS children from both healthy children and children with several other genetic syndromes. Also, the DCNN-Arcface model is more suitable for identifying NS. Our study offers compelling evidence that the DCNN-Arcface model can improve the diagnostic rate of NS. Our results also indicate that the DCNN-Arcface model can be adapted to detect other heterogeneous genetic syndromes.

The DCNN-Arcface model also outperformed two traditional machine learning methods. The AUC of DCNN-Arcface model was 0.9797 ± 0.0055 when discriminating NS patients from healthy children, while the AUCs of the two traditional machine learning models in the same task were 0.9031 ± 0.0064 and 0.8669 ± 0.0035, respectively. There are several possible explanations for this result. First, the DCNN-based model has many more parameters than the machine learning-based models, leading to better representation ability for fitting into the unknown function of input images and output prediction. The deep structure also enables the network to extract latent features layer-by-layer from raw images of NS patients’ faces. Moreover, not all selected features are informative for NS identification with the traditional machine learning method, and other useful features may be lost. In contrast, the DCNN performs feature extraction and classification in an end-to-end manner, which avoids any manual feature-selection bias. Finally, the ArcFace loss function increases the neural network’s discriminative power for different classes, while the loss function for the traditional machine learning model does not provide this benefit (Wang et al., 2018; Deng et al., 2019; Zhang et al., 2020).

The primary limitation of this study is that there were a limited number of dysmorphic facial photographs of NS patients. This might have led to over-fitting. In the future, we will conduct a multicenter study to collect more photographs, and we will explore the use of data augmentation methods, such as the generative adversarial networks, to generate more face images of NS patients.

## Conclusion

In conclusion, this study illustrated that the proposed facial recognition model based on DCNN and Arcface loss function could play a prominent role in NS diagnosis. The results highlight the feasibility of facial recognition technology to identify NS in clinical practice.

## Data Availability Statement

The raw data supporting the conclusions of this article will be made available by the authors, without undue reservation.

## Ethics Statement

The studies involving human participants were reviewed and approved by Research Ethics Committee of Guangdong Provincial People’s Hospital. Written informed consent to participate in this study was provided by the participants’ legal guardian/next of kin. Written informed consent was obtained from the minor(s)’ legal guardian/next of kin for the publication of any potentially identifiable images or data included in this article.

## Author Contributions

S-SW: conceptualization, funding acquisition, project administration, supervision, writing – review and editing, and funding. HY and X-RH: data curation, methodology, validation, writing – original draft. HY, X-RH, LS, DH, Y-YZ, YX, HL, M-YL, LW, and D-PL: resources. HY: software. All authors contributed to the article and approved the submitted version.

## Conflict of Interest

The authors declare that the research was conducted in the absence of any commercial or financial relationships that could be construed as a potential conflict of interest. The reviewer, YS, declared a shared affiliation with one of the author, X-RH, to the handling editor at the time of review.

## Supplementary Material

Supplementary Table Characterization of the mixed dysmorphic genetic syndromes dataset.
