# Supplementary material for: Automated Facial Recognition for Noonan Syndrome Using Novel Deep Convolutional Neural Network With Additive Angular Margin Loss
Source: Front Genet. 2021 Jun 7;12:669841. doi: 10.3389/fgene.2021.669841 (PMC8215580; doi:10.3389/fgene.2021.669841)
Supplement: Supplementary Table — Characterization of the mixed dysmorphic genetic syndromes dataset. [file Table_1.DOCX]

Supplementary Material

# **Supplementary Table 1：**Characterization of the mixed dysmorphic genetic syndromes dataset.

| **Syndorme** | **Male** | **Female** |
| --- | --- | --- |
| Williams-Burden Syndrome | 56 | 16 |
| Microdeletion Syndrome | 8 | 6 |
| Down Syndrome | 6 | 6 |
| Microduplication Syndrome | 6 | 4 |
| Loeys- Dietz Syndorme | 3 | 1 |
| Digeroge Syndorme | 1 | 2 |
| Alagille Syndrome | 1 | 2 |
| Andersen Syndrome | 0 | 1 |
| Helsmoortel-van der Aa Syndrome | 1 | 0 |
| Marfan Syndrome | 0 | 1 |
| Myhre Syndrome | 0 | 1 |
| Barth Syndrome | 1 | 0 |
| Wolf-Hirschhorn Syndrome | 0 | 1 |
| Coffin-siris Syndrome | 0 | 1 |
| Stickler Syndrome | 1 | 0 |
| Cornelia de Lange Syndrome | 0 | 1 |
| Holf-oram Syndrome | 0 | 1 |
| Costello Syndrome | 1 | 1 |
